# LC-MS/MS assisted pharmacokinetic and tissue distribution study of ropivacaine and 3-OH-ropivacaine on rats after plane block anesthesia

**DOI:** 10.3389/fphar.2024.1494646

**Published:** 2025-01-07

**Authors:** Mihaela Butiulca, Lenard Farczadi, Silvia Imre, Camil Eugen Vari, Laurian Vlase, Bogdan Cordos, Leonard Azamfirei, Alexandra Elena Lazar

**Affiliations:** ^1^ Department of Anaesthesiology and Intensive Care Medicine, Faculty of General Medicine, George Emil Palade University of Medicine, Pharmacy, Science, and Technology of Targu Mures, Târgu Mureș, Romania; ^2^ Department of Anaesthesiology and Intensive Care Medicine, Emergency County Hospital, Târgu Mureș, Romania; ^3^ Chromatography and Mass Spectrometry Laboratory, Center for Advanced Medical and Pharmaceutical Research, George Emil Palade University of Medicine, Pharmacy, Science, and Technology of Targu Mures, Târgu Mureș, Romania; ^4^ Department of Analytical Chemistry and Drug Analysis, Faculty of Pharmacy, George Emil Palade University of Medicine, Pharmacy, Science, and Technology of Targu Mures, Târgu Mureș, Romania; ^5^ Department of Pharmacology and Clinical Pharmacy, Faculty of Pharmacy, George Emil Palade University of Medicine, Pharmacy, Science, and Technology of Targu Mures, Târgu Mureș, Romania; ^6^ Department of Pharmaceutic Technology and Biopharmacy, “Iuliu Hațieganu” University of Medicine and Pharmacy, Cluj-Napoca, Romania; ^7^ Department of Experimental and Imaging Studies, Center for Advanced Medical and Pharmaceutical Research, George Emil Palade University of Medicine, Pharmacy, Science, and Technology of Targu Mures, Târgu Mureș, Romania

**Keywords:** ropivacaine, pharmacokinetics, LC-MS/MS, rats, HPLC

## Abstract

Knowledge of drug pharmacokinetics and tissue distribution is precious for ensuring patient safety and optimizing treatments. The varied use of local anesthetics, as well as the fact that anesthetics can sometimes have low therapeutic indices and numerous adverse drug reactions, makes any novel pharmacokinetics information valuable. The present manuscript describes a pharmacokinetic study of ropivacaine carried out after plane block anesthesia on an animal model, using high sensitivity, accurate, and precise LC-MS/MS bioanalysis. Both plasmatic concentrations and tissue distribution of ropivacaine and its primary active metabolite were determined. The results showed a tissue affinity of the anesthetics, a clearance of ropivacaine mainly by hepatic metabolism, and the final, mainly renal excretion of the hydroxylated metabolite. While the results cannot simply and directly be transposed to human pharmacokinetics, they offer a valuable basis for future studies and can contribute to a better understanding of the bioavailability and toxicology of the widely used modern anesthetic.

## 1 Introduction

In the perioperative period, ensuring the comfort and safety of the patient is crucial for the recovery process. This is one of the reasons why regional anesthesia, particularly the use of ropivacaine, is widely practiced today. Ropivacaine is a popular local anesthetic known for its prolonged effects and superior safety profile to other long-acting local anesthetics such as bupivacaine (T3DB, 2024). However, it is essential to note that while ropivacaine is safer, its toxicity level is not negligible, and a better understanding of its pharmacokinetic profile could enhance its safety.

Ropivacaine is a pure S-enantiomer metabolized by cytochrome P450 into several metabolites, including 3-OH ropivacaine, 2′,6′-pipecoloxylidide, 4-OH-ropivacaine, and 2-OH-methyl ropivacaine (Ekstrom and Gunnarsson, 1996). The plasma concentration of ropivacaine is influenced by factors such as the total dose, method of administration, patient’s hemodynamic status, and vascularity of the infiltration site (Simpson et al., 2005).

Accurately measuring medicines or endogenous substances in biological samples is vital for medical and pharmaceutical research (Olivares-Morales et al., 2015). Bioavailability is critical for determining drug safety and efficacy. Biomonitoring through various techniques, including chromatography, is utilized for research, drug development, and regulatory evaluation of safety and efficacy (Nikolin et al., 2004; Mostafa et al., 2022).

While several studies have reported plasma concentration measurements, more information must be given regarding determining ropivacaine concentration in tissues using HPLC LC-MS/MS. Understanding the tissue distribution of ropivacaine is crucial for assessing its toxicological profile, and the tissue/plasma ratio may offer insight into the risk of accumulation and potential adverse events from systemic exposure to ropivacaine (Wang et al., 2020).

This study aims to introduce an advanced methodology for determining the concentration of ropivacaine and its metabolites in various matrices in rats, including brain tissue, liver, lung, kidney, heart, fatty tissue, and plasma.

## 2 Methods

Due to ethical concerns surrounding the collection of human tissue samples, the study utilizes an animal model (rats) to investigate the tissue distribution pattern of ropivacaine administered under regional anesthesia. The rat model is selected for its anatomical, metabolic, and genetic similarity to humans and ease of manipulation. The results from this study may serve as a basis for clinical pharmacokinetic and toxicological assessments and aid in preventing systemic toxicity resulting from accidental exposure to high plasma concentrations of ropivacaine.

The study was conducted in compliance with ethical standards. Approval was obtained from the University (1893/19.10.2022) and local veterinary services by European norms (55/06.03.2023).

### 2.1 Experimental protocol

**FIGURE 1:**
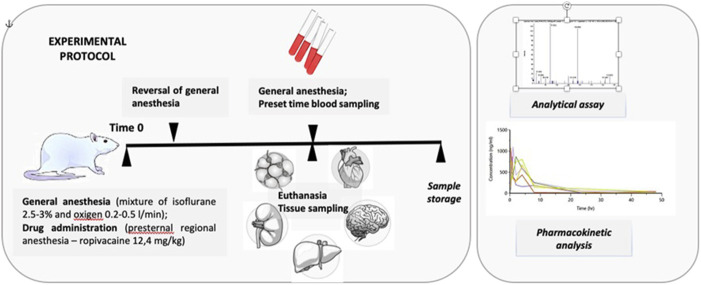
Experimental protocol.

The pharmacokinetic study involved 54 white Wistar rats, both male and female, aged between 4 months and 1 year. They were divided into groups of 6 animals, consisting of three males and three females. Blood samples were collected at various intervals: 15, 30, 45, 60, 120, 240, 480, 1,440, and 2,880 min. The minimum number of animals per group was determined considering the high inter-individual variability of the pharmacokinetic parameters and ethical concerns. Ropivacaine concentrations were measured in tissue samples of the liver, lung, brain, kidney, heart, adipose tissue, muscle tissue, and plasma samples (see Figure 1).

### 2.2 Inclusion and exclusion criteria

Clinically healthy male and female rats were included in the study. Exclusion criteria were rats younger than 4 months and older than 1 year to more accurately mimic the human pharmacokinetic profile and enhance the reproducibility of the interindividual metabolism process.

### 2.3 Animal care

The animal subjects were obtained from the animal facility of George Emil Palade University of Medicine, Pharmacy, Sciences, and Technology in Târgu Mureș, Romania. They were individually weighed, housed in isolated cages, and maintained at 22°C and 50% humidity. The animals were kept on a standard sleep-wake cycle of 12 h each. Their weight ranged from 220 g to 450 g. The animals were individually anesthetized in the induction chamber using a gas mixture of 5% Isoflurane and oxygen at a rate of 0.8 L/min. Anaesthesia was then maintained using a mask containing 2.5%–3% isoflurane and oxygen at 0.2–0.5 L/min.

For regional anesthesia, a 26 G needle was used to perform a parasternal puncture. This site was chosen to simulate a pectoral nerve block in human subjects. The amount of local anesthetic administered was equivalent to 2 mg.kg-1 body weight for human subjects, which translated to 12.4 mg.kg-1 body weight in rats based on the calculated HED (human equivalent dose) value derived from the ratio of body surfaces (Nair and Jacob, 2016). A mixture of 1% lidocaine and ropivacaine in equal parts was used for administration.

General anesthesia was reversed after administering the local anesthetic, and the animals were placed in cages. Throughout the study, they had unrestricted access to water and food.

After each group’s scheduled study time, they were returned to the induction room and put under general anesthesia using the same gas mixture. Isoflurane 5% was used for maintenance.

The procedure involved a bilateral clamshell thoracotomy to expose the thoracic cavity. Then, a 20-gauge needle was used to perform an intracardiac puncture and extract a volume of blood ranging from 10 to 20 mL based on sex, age, and weight. The animals were kept under continuous anesthesia until they succumbed to hypovolemic shock. The absence of cardiac activity confirmed death.

Following this, 5 mL of blood with a clot activator and gel separator was stored in vacutainers and subjected to primary processing. The blood sample was centrifuged at 1300 G for 10 min, and the resulting serum was stored in 1.5 mL Eppendorf tubes at −80°C for future use.

Tissue samples were collected from the liver, lung, brain, kidney, heart, adipose tissue, and muscle tissue and stored at −80°C in self-standing polypropylene tubes. After all the samples were collected, the animals were cremated to neutralize hazardous waste.

### 2.4 LC-MS/MS bioanalysis

For the LC-MS/MS bioanalysis of the samples, plasma samples were deproteinized before analysis using protein precipitation with acetonitrile. Specifically, 200 μL of plasma sample was mixed with 100 μL of internal standard and 500 μL of acetonitrile. The mixture was vortexed for 2 min and centrifuged for 3 min at 1300 G, and the supernatant was analyzed after injection into the LC-MS/MS system.

The tissue samples (brain, heart, lung, kidney, liver, muscle) were processed individually. First, they were weighed, then ground and diluted with 3 mL of ethyl acetate, ultrasonicated for 5 min, and vortexed for 2 min. Next, 2.5 mL of the ethyl acetate-containing organ residue was centrifuged for 10 min at 2000 G. The supernatant was transferred to a clean polypropylene tube and evaporated at ambient temperature under vacuum for 3 h. The dry residue left after processing was reconstituted in 0.7 mL of acetonitrile: water (5:2, v/v) mixture and 0.1 mL internal standard (0.5 μg.mL-1) solution. The reconstituted sample solution was placed in HPLC vials and injected into the LC-MS/MS system.

A weighted amount of adipose tissue was shredded and ground with 2 mL of hexane for the adipose tissue samples. The resulting mixture was then transferred to polypropylene tubes and ultrasonicated for 2 h. Following this, 1.4 mL of acetonitrile: water (5:2, v/v) mixture was added, and the mixture was vortexed for 1 h at 800 rpm. A volume of 0.7 mL of the polar phase (acetonitrile: water mixture) was transferred to HPLC vials, and 0.1 mL of internal standard (0.5 μg.mL-1) was added and mixed in. Finally, the sample was injected into the LC-MS/MS system.

### 2.5 Method validation

**FIGURE 2:**
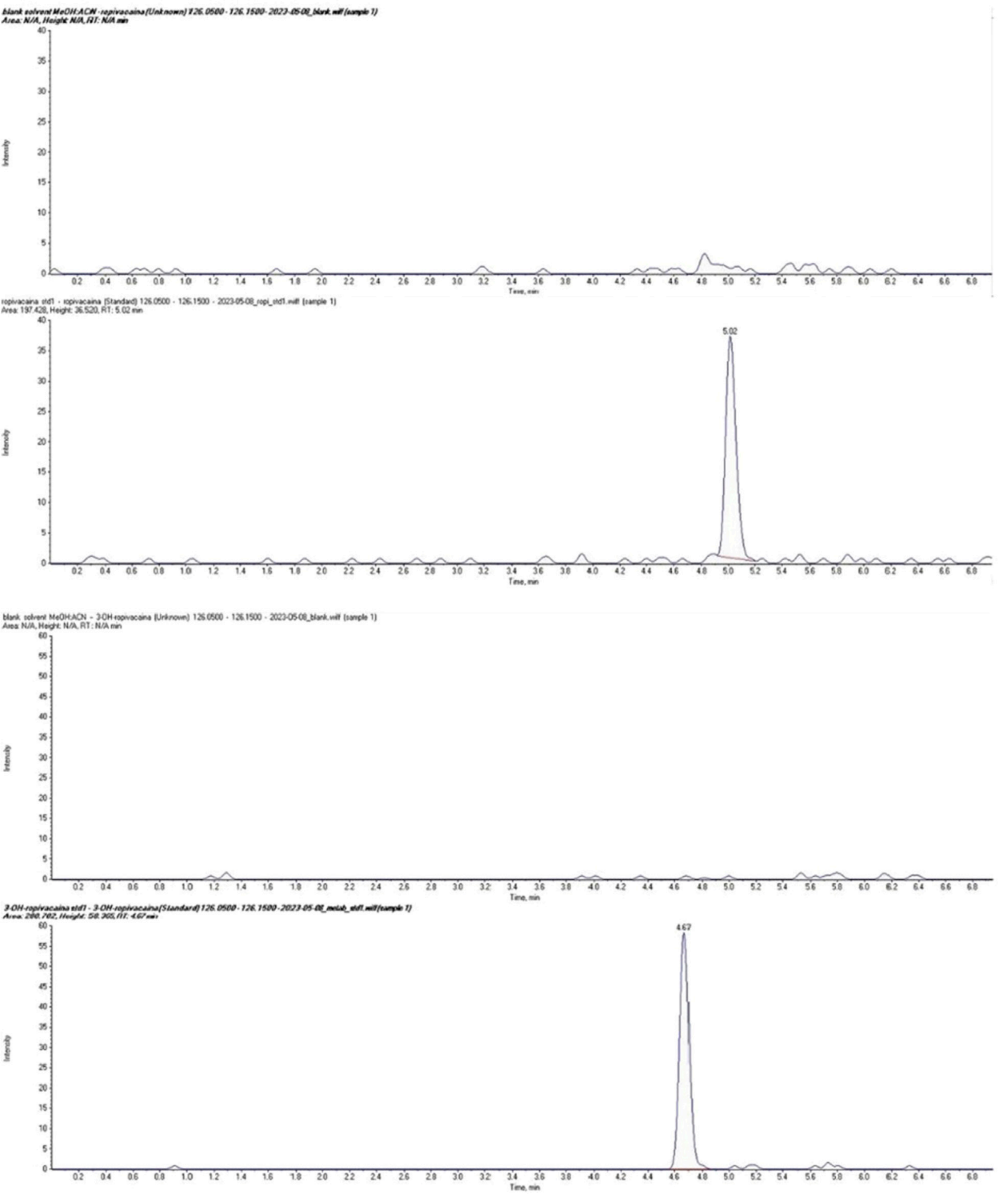
Chromatograms of ropivacaine (top) and 3-OH ropivacaine (bottom) in standard solution compared to blank samples.

**FIGURE 3:**
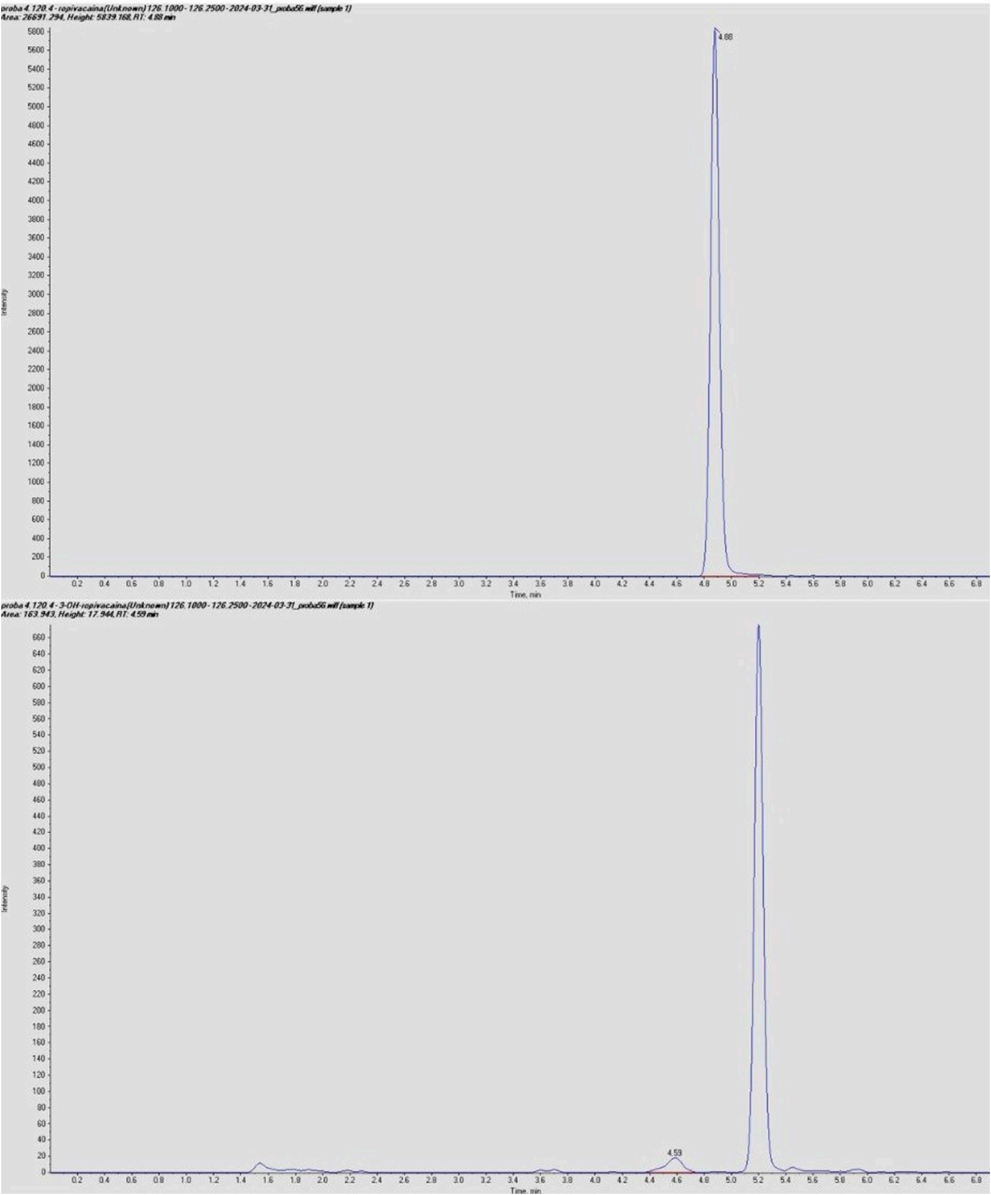
Chromatograms of ropivacaine and 3-OH ropivacaine in plasma samples.

The LC-MS/MS method was validated according to Food and Drug Administration (FDA, 2018) and European Medicines Agency (EMA, 2011) guidelines for bioanalytical method validation, covering parameters such as accuracy, precision, linearity, selectivity, sensitivity, matrix effect, and analyte recovery. The complete method development and validation procedure and the results have been previously published (Butiulca et al., 2023). Typical chromatograms for ropivacaine and 3-OH-ropivacaine in blank, standard, and plasma samples are shown in Figures 2, 3.

### 2.6 Equipment used

The LC-MS/MS system used for analyte quantification consisted of a PerkinElmer (United States) FX-10 HPLC coupled to an AB Sciex (United States) Triple TOF 4600 MS. Other equipment used included an Eppendorf (Germany) 5430R centrifuge, a Radwag (Poland) XA 52.3Y analytical balance, a Velp Scientifica (Italy) ZX4 vortex mixer, a JP Selecta (Spain) Ultrasons H-D ultrasonic bath, and a Thermo (United States) Speed Vac sample evaporator and concentrator.

### 2.7 Analysis of primary data

The quantitation of ropivacaine and 3-OH-ropivacaine using LC-MS/MS was performed with a linear fit calibration curve and 1/y^2 weighting in AB Sciex Analyst software. The results were calculated in ng.ml-1 based on the concentration of the calibration standard solutions for all biological sample types (EMA, 2011). These results were used for statistical and pharmacokinetic analysis of plasma samples. For tissue samples, the analyte concentrations in the final sample solution were correlated with the amount of tissue (brain, heart, lung, kidney, liver, adipose, muscle) and expressed as ng.g-1 of tissue. All statistical analysis for tissue samples was performed based on these quantitation results.

### 2.8 Pharmacokinetic analysis

The results data was analyzed using Phoenix WinNonlin pharmacokinetic software to perform descriptive statistics and pharmacokinetic analysis. This involved calculating primary pharmacokinetic parameters and using non-compartmental analysis to estimate quantities of interest directly from the concentration-time curve.

## 3 Results

**FIGURE 4:**
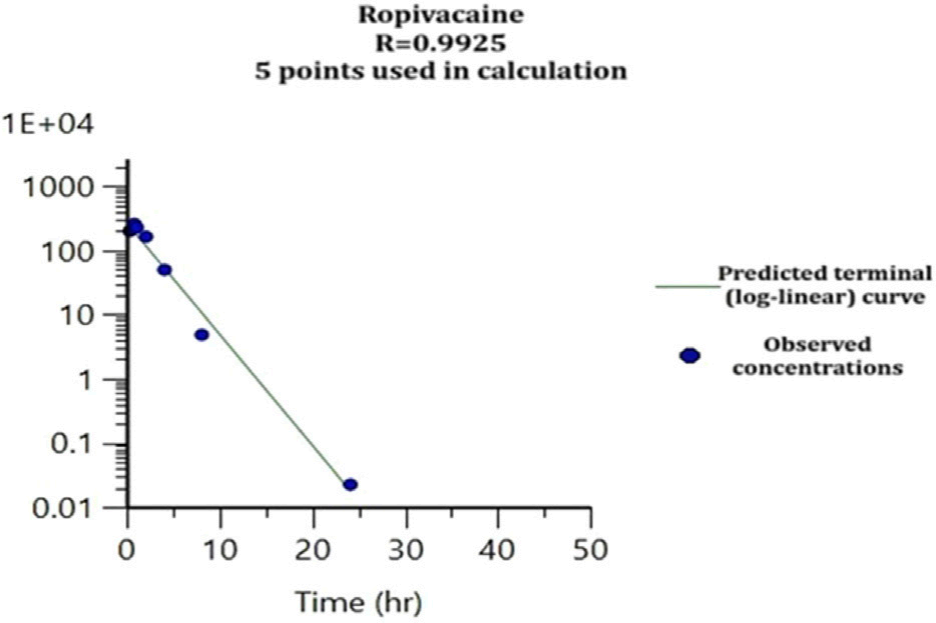
Elimination curve of ropivacaine from plasma.

The concentrations of ropivacaine and 3-OH-Ropivacaine in plasma indicate quick absorption into the bloodstream and slow, steady metabolism and elimination (see Figure 4). The concentrations in organ tissues show an uneven distribution among the different organs. As expected, the highest concentrations of ropivacaine are found in the puncture area (lungs) and the kidneys. On the other hand, the metabolite has lower bioavailability in the plasma and is mainly distributed in the liver and kidneys. The mean concentrations measured are presented in Tables 1, 2.

**TABLE 1:** The mean concentration of ropivacaine in organ tissue and plasma.

Time	Mean concentration, ropivacaine [ng.g^-1^ organ tissue or.ml^-1^ plasma]							
	Liver	Lung	Heart	Brain	Muscle	Kidney	Adipose	Plasma
15 min	94.51 ± 75.23	728.91 ± 220.87	307.41 ± 88.91	444.66 ± 105.61	139.83 ± 74.39	816.20 ± 240.09	393.81 ± 354.13	206.73 ± 47.59
30 min	162.15 ± 131.28	786.71 ± 263.25	393.66 ± 274.68	523.92 ± 198.64	212.78 ± 92.82	850.26 ± 461.30	593.33 ± 241.00	219.34 ± 58.34
45 min	153.73 ± 80.10	897.76 ± 231.44	375.04 ± 302.95	439.34 ± 136.94	186.49 ± 43.65	469.74 ± 126.06	624.26 ± 200.07	268.22 ± 24.17
60 min	107.46 ± 61.24	954.63 ± 544.55	337.50 ± 143.08	412.92 ± 199.60	76.71 ± 40.87	403.45 ± 166.48	526.72 ± 286.31	234.19 ± 90.04
120 min	90.99 ± 55.15	699.58 ± 201.24	200.63 ± 127.56	257.73 ± 84.14	69.27 ± 35.99	312.73 ± 139.23	467.84 ± 275.52	169.16 ± 63.77
240 min	59.76 ± 44.46	243.84 ± 185.77	108.33 ± 75.73	418.33 ± 292.83	150.01 ± 125.18	313.88 ± 341.99	540.51 ± 222.17	51.52 ± 35.32
480 min	3.29 ± 1.71	77.29 ± 85.25	13.40 ± 9.39	11.49 ± 6.98	6.36 ± 4.77	17.40 ± 12.03	174.04 ± 88.37	5.02 ± 2.63
1,440 min	0.00 ± 0.00	2.59 ± 4.66	0.58 ± 0.65	1.24 ± 1.96	0.13 ± 0.20	0.00 ± 0.00	35.20 ± 39.72	0.02 ± 0.06
2,880 min	0.29 ± 0.64	2.15 ± 4.49	0.34 ± 0.63	0.75 ± 1.07	0.08 ± 0.21	1.59 ± 2.54	25.72 ± 17.48	0.00 ± 0.00

**TABLE 2:** The mean concentration of 3-OH-ropivacaine in organ tissue and plasma.

Time	Mean concentration, 3-OH-ropivacaine [ng.g^-1^ organ tissue or.ml^-1^ plasma]							
	Liver	Lung	Heart	Brain	Muscle	Kidney	Adipose	Plasma
15 min	729.49 ± 439.89	83.00 ± 31.04	25.29 ± 11.28	28.67 ± 17.48	13.73 ± 20.08	416.49 ± 238.57	0.00 ± 0.00	15.35 ± 8.68
30 min	1143.62 ± 533.27	89.82 ± 34.40	19.90 ± 8.31	22.50 ± 10.40	8.75 ± 5.21	251.22 ± 201.97	1.04 ± 2.55	12.67 ± 5.88
45 min	923.42 ± 339.84	137.97 ± 66.41	38.44 ± 19.33	27.69 ± 17.63	21.89 ± 8.65	210.04 ± 105.94	2.69 ± 4.12	30.20 ± 8.85
60 min	1092.17 ± 436.48	111.34 ± 74.44	23.59 ± 14.18	18.88 ± 18.27	0.08 ± 0.19	60.10 ± 20.50	0.00 ± 0.00	18.05 ± 12.60
120 min	226.62 ± 157.52	19.17 ± 23.75	2.82 ± 4.38	0.96 ± 1.49	1.41 ± 3.46	21.07 ± 31.73	1.27 ± 3.12	20.94 ± 10.84
240 min	435.10 ± 232.11	46.16 ± 30.27	25.32 ± 11.45	73.75 ± 47.47	22.58 ± 17.56	141.04 ± 125.55	3.26 ± 4.71	17.60 ± 9.41
480 min	306.04 ± 174.35	38.00 ± 37.43	15.75 ± 8.91	3.29 ± 1.32	5.06 ± 12.15	44.46 ± 14.99	10.61 ± 2.48	4.39 ± 2.51
1,440 min	45.19 ± 38.26	3.93 ± 7.86	4.92 ± 10.32	2.02 ± 4.63	0.00 ± 0.00	20.40 ± 26.02	0.00 ± 0.00	3.84 ± 6.82
2,880 min	36.90 ± 38.38	2.35 ± 3.68	5.06 ± 12.39	0.00 ± 0.00	0.00 ± 0.00	4.45 ± 4.01	0.00 ± 0.00	0.00 ± 0.00

**FIGURE 5:**
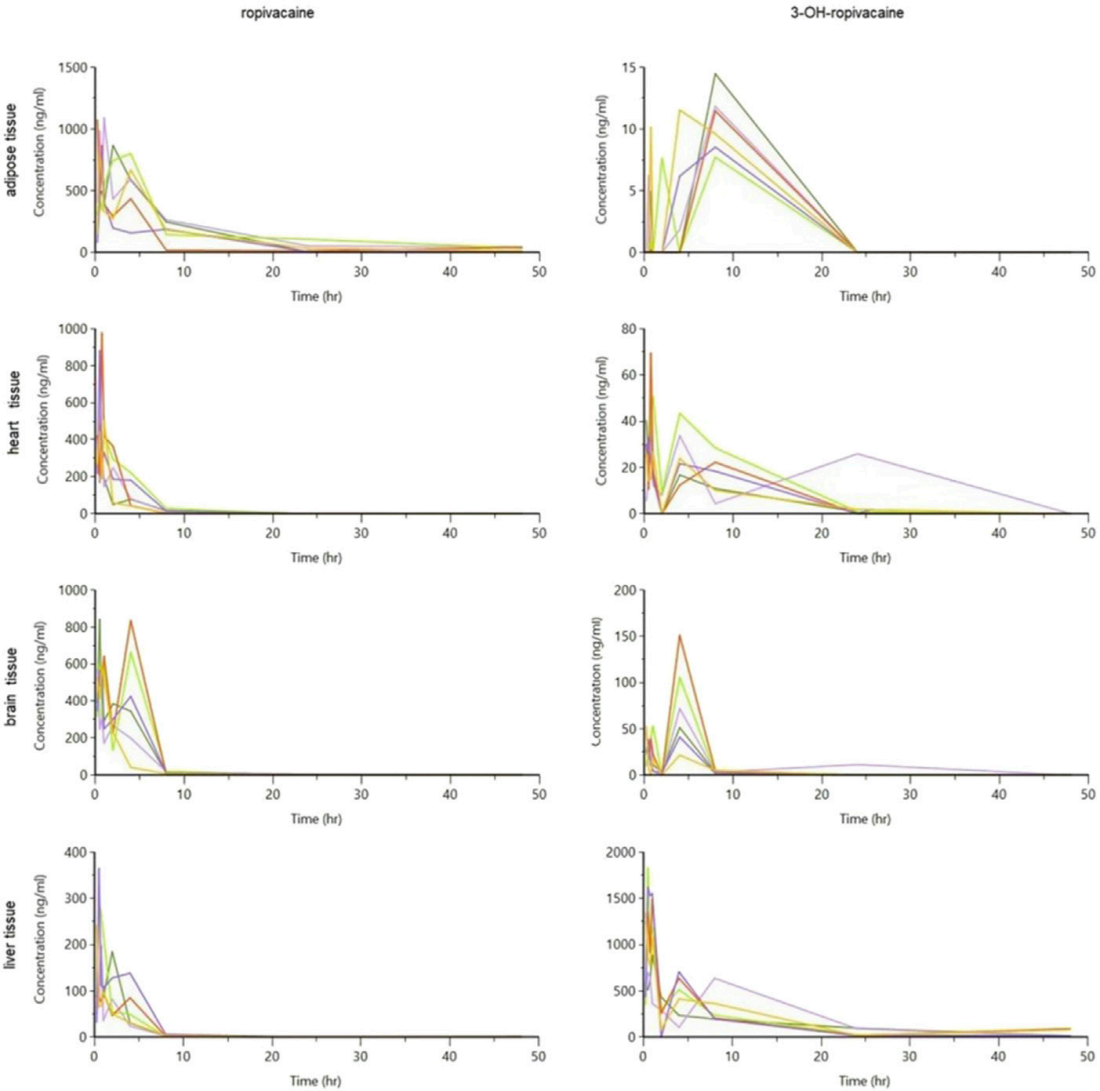
Spaghetti plot of calculated tissue concentrations of ropivacaine and 3-OH-ropivacaine (concentrations are expressed in ng/g tissue, and time in hours).

**FIGURE 6:**
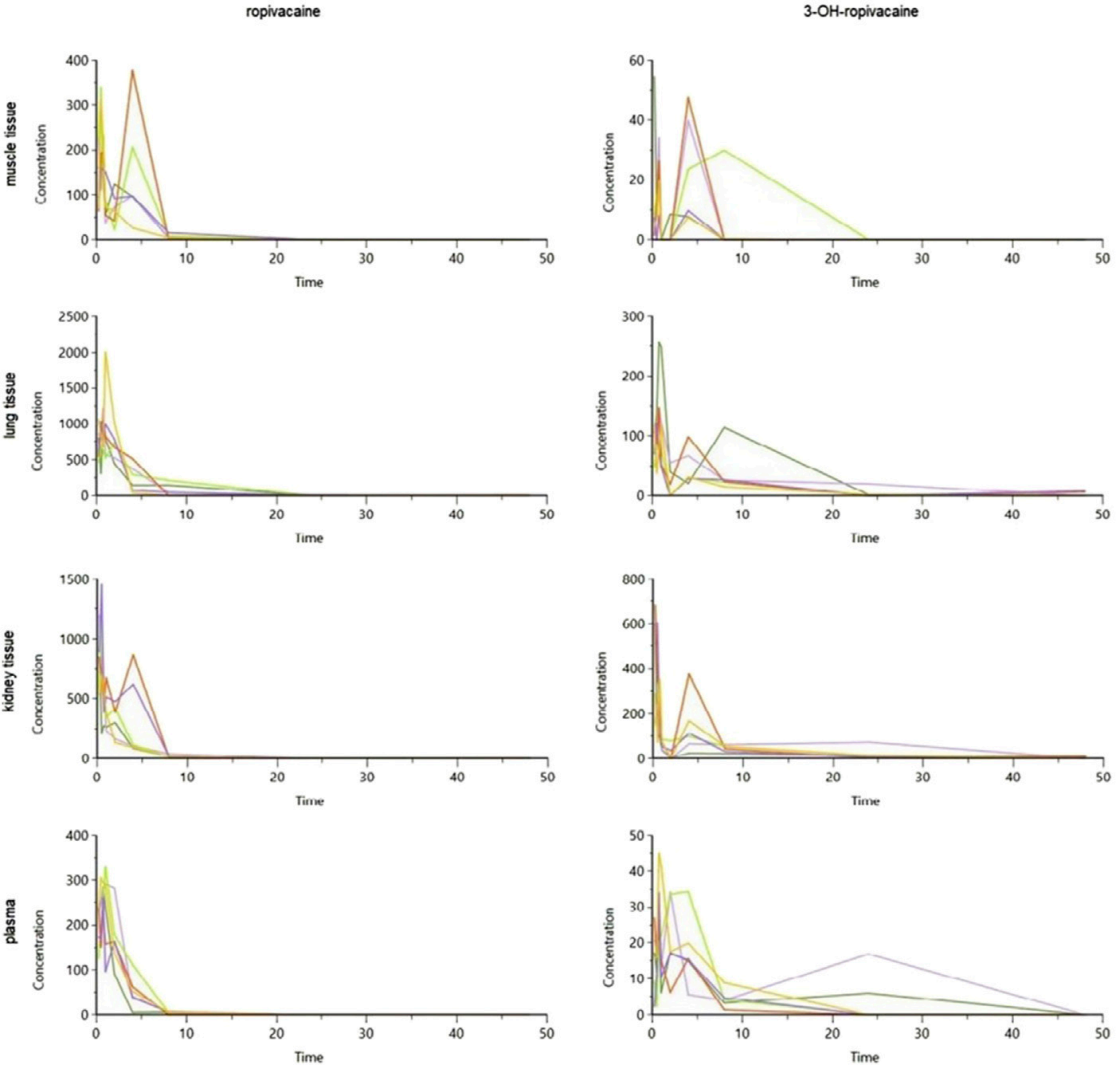
Spaghetti plot of calculated tissue and plasma concentrations of ropivacaine and 3-OH-ropivacaine (concentrations are expressed in ng/g tissue, and ng/mL for plasma, and time in hours).

The tissue and plasma concentrations were plotted separately for each type of organ and ropivacaine and its primary metabolite, and they were overlayed in spaghetti plots in Figures 5, 6.

The mean pharmacokinetic parameters after non-compartmental analysis are presented in Tables 3, 4.

**TABLE 3:** The mean pharmacokinetic parameters after non compartmental analysis for ropivacaine.

Substance	Organ	Cmax (ng.mL^-1^)	Tmax (h)	AUC (hr*ng ml^-1^)	Elimination rate constant (1.h^-1^)	Terminal half-life (h)	Volume of distribution (L.kg^-1^)	Total body clearance (L.h-1.kg-1)
Ropivacaine	Plasma	268.22	0.75	778.95	0.40	1.74	38.61	15.41
	Adipose	624.26	0.75	5808.40	0.12	5.69	16.92	2.06
	Heart	393.66	0.50	1255.54	0.28	2.50	34.52	9.56
	Brain	523.92	0.50	2400.40	0.26	2.65	19.14	5.00
	Liver	162.15	0.50	521.93	0.12	5.65	186.68	22.92
	Muscle	212.78	0.50	803.76	0.17	4.05	87.31	14.93
	Lung	954.63	1.00	3831.41	0.12	5.65	25.50	3.13
	Kidney	850.26	0.50	2389.96	0.11	6.04	43.58	5.00

**TABLE 4:** The mean pharmacokinetic parameters after non compartmental analysis for 3-OH-ropivacaine.

Substance	Organ	Cmax (ng.mL^-1^)	Tmax (h)	AUC (hr*ng ml^-1^)	Elimination rate constant (1.h^-1^)	Terminal half-life (h)	Volume of distribution (L.kg^-1^)	Total body clearance (L.h-1.kg-1)
3-OH-Ropivacaine	Plasma	30.20	0.75	230.75	0.07	9.47	720.78	52.78
	Adipose	10.61	8.00	118.69	-	-	-	-
	Heart	38.44	0.75	432.32	0.04	19.63	627.83	22.17
	Brain	73.75	4.00	327.54	0.14	5.05	278.22	38.17
	Liver	1,143.62	0.50	7434.06	0.05	13.15	28.64	1.51
	Muscle	22.58	4.00	131.64	0.37	1,85	306.65	114.64
	Lung	137.97	0.75	801.42	0.07	9.72	204.17	14.55
	Kidney	416.49	0.25	1671.65	0.06	11.94	121.83	7.07

**FIGURE 7:**
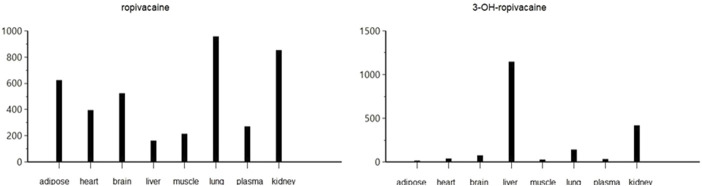
Maximum concentration (Cmax) of ropivacaine and 3-OH-ropivacaine in tissues and plasma (ng.mL^-1^).

**FIGURE 8:**
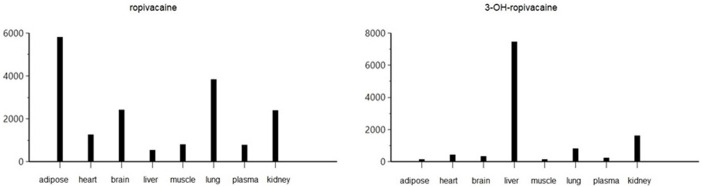
Area under the curve to the last measurable concentration (AUClast) of ropivacaine and 3-OH-ropivacaine in tissues and plasma (ng.mL^-1^).

**FIGURE 9:**
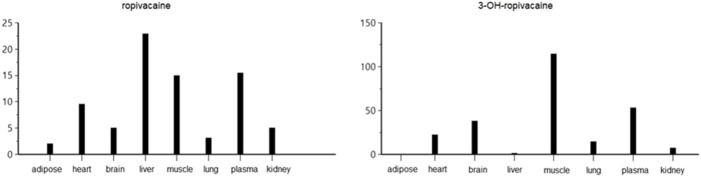
Clearance (CL) of ropivacaine and 3-OH-ropivacaine from tissues and plasma (L.h-1. kg-1).

The pharmacokinetic analysis of the data indicates that the highest concentration of ropivacaine is reached within the first hour after the plane block procedure, even in adipose tissue. Afterward, a rapid metabolism process begins in the liver. The metabolite reaches its highest concentration within the first hour after administration for plasma, heart, lung, and liver tissues. However, the maximum concentrations of 3-OH-ropivacaine in brain, muscle, and adipose tissue are achieved after a few hours, indicating a slow accumulation in these tissues. While 3-OH-ropivacaine distributes predictably throughout different organ tissues, with the highest concentrations in the liver and kidneys, ropivacaine tends to accumulate in adipose tissue (as indicated by a high AUC) and show very high concentrations in lung and brain tissues (see Figures 7–9).

The study emphasizes the significance of understanding the way ropivacaine is processed in the body and how much of it is available for use, especially during plane block anesthesia. The study, which was conducted on rats to simulate the plane block procedure performed on humans, indicates the importance of being cautious when administering ropivacaine as an anesthetic to prevent any toxic effects and harm to patients. Although the study was carried out on an animal model and cannot directly be transposed to a human PK model, it does provide some important information which can help in modelling a human study model. At the same time, the limited data available on PK models, both animal and human, underline the importance of more future research on this topic, research for which the current manuscript can be an important starting point and source of information. As of now, there is minimal data available for both approved and off-label use of ropivacaine, further highlighting the importance of data obtained in both animal and human PK studies of this substance.

## 4 Discussions

Toxicity of local anesthetics is due to systemic exposure to the active substance, depending on the mode of administration (epidural infusion, continuous peripheral nerve anesthesia or intermittent administration, bolus administration), total cumulative dose, local blood flow, and individual characteristics influencing distribution (especially adipose tissue/muscle ratio) and clearance (kidney and liver functional status). Given the marked lipophilicity of the molecule, overdose diagnosed promptly based on clinical signs benefits from the antidote effect of lipid infusions and supportive therapy (Long et al., 2022). In the case of nerve block, the technique used can significantly influence both the plasma ropivacaine level and the Tmax value, as described for brachial plexus block (Rettig et al., 2007); also, the concomitant administration of adrenaline, by decreasing local blood flow, considerably modifies the systemic exposure to the anesthetic (Kazune et al., 2023).

There are human pharmacokinetic data to assess the dose-toxicity relationship, and the threshold value for the toxicity threshold remains controversial: 3.4 µg.mL-1 (Zaballos et al., 2023) or 2.2 μg.mL-1 (Kazune et al., 2023; Brydone et al., 2015). A study involving 18 patients demonstrated that ropivacaine doses at the higher end of the clinical range (800 mg/day) did not result in the symptoms of LAST, despite some patients having ropivacaine concentrations exceeding the toxic threshold (Matsota et al., 2024). Also, after long-term continuous epidural administration, plasma concentration values above this threshold are described without signs of toxicity, which seems to confer safety and innocuousness to ropivacaine treatment (Wiedemann et al., 2000).

The most severe side effect described in acute overdose is ventricular fibrillation preceded by convulsions after accidental intravenous administration (Gielen et al., 2005). Seizure occurrence after accidental intravenous injection also benefited from estimating the maximum plasma level (above 5 μg.mL-1) based on a two-compartment model following serially measured plasma concentrations after administration (Müller et al., 2001). However, seizures can also occur at plasma concentrations in the safe zone (2.13 μg.mL-1), according to a case report by Satsuma et al. (Satsumae et al., 2008). Signs of nervous system damage in overdose (convulsions) are more common than cardiovascular disorders. Even at values above the toxicity threshold, hemodynamic parameters are not affected, but QTc interval prolongation is described in an animal model (Zaballos et al., 2023); this fact may raise the problem of drug interactions.

An animal model somewhat like ours, but using piglets as the target species, followed the pharmacokinetics of ropivacaine after a serratus intercostal fascial plane block but aimed to monitor potential effects on electrophysiological and hemodynamic parameters (Zaballos et al., 2023). While the number of studies researching the pharmacokinetics of ropivacaine is limited, their importance must be recognized. As one of the essential local anesthetics, any new information on the absorption and distribution throughout the body, as well as the metabolization and elimination of ropivacaine and its primary active metabolite, is of value for future research and optimization of therapeutic use.

Although the number of studies investigating the pharmacokinetics of ropivacaine is relatively limited, their importance must be recognized. As one of the essential local anesthetics, any new information on the absorption and distribution throughout the body, as well as the metabolization and elimination of ropivacaine and its primary active metabolite, is of value for future research and optimization of therapeutic use. Enhancing our knowledge of the pharmacokinetics of ropivacaine is crucial for improving patient safety, especially regarding potential systemic side effects such as cardiovascular or central nervous system toxicity.

The first study describing tissue distribution of ropivacaine and 3-OH-ropivacaine after plane block administration, the results of our research give valuable information on the distribution of ropivacaine throughout the different organs and can form the basis for further research and therapeutic drug monitoring for patients in critical situations where an accurate drug level can improve patient recovery reduce adverse effects without compromising the efficacy of the anesthetic procedure or causing discomfort, or even pain, to patients.

The study’s limitations include the low number of animals due to ethical aspects. The design of the study requires the sampling of organs for tissue analysis. Individual pharmacokinetics could not be done in each animal, so we had to limit ourselves to different animals at each sampling time, which reduced the correlation with natural pharmacokinetics made for each individual. Furthermore, altough helpful as a basis for human PK studies, the results of this study cannot be directly extrapolated to a human model, but rather serve as a starting point and source of information in certain regards (possible distribution, clearance and metabolic pathways, *etc.*).

## 5 Conclusion

Our study examined how ropivacaine is distributed in the tissues of an animal model. This is the first study to describe a tissue distribution and pharmacokinetics of ropivacaine and 3-OH-ropivacaine, using animal model. We found that the drug has a greater affinity for specific tissues (brain, muscle, adipose tissue, lung, kidney) than plasma. This was indicated by the high volume of distribution and the higher concentrations of the drug in these organs compared to plasma. The drug is mainly broken down by the liver, with hepatic clearance values about four times higher than renal clearance. Therefore, the drug is mainly excreted in the urine as a primary metabolite produced by liver processing. While we should be careful when applying these findings to humans, the information we gathered about plasma levels, tissue distribution, and differential clearance of ropivacaine after systemic exposure is valuable for estimating drug toxicity following regional anesthesia.

## Data Availability

The raw data supporting the conclusions of this article will be made available by the authors, without undue reservation.
